# A Computational Model for the PLP-Dependent Enzyme Methionine *γ*-Lyase

**DOI:** 10.3389/fmolb.2022.886358

**Published:** 2022-04-26

**Authors:** Xingyu Chen, Pierre Briozzo, David Machover, Thomas Simonson

**Affiliations:** ^1^ Laboratoire de Biologie Structurale de la Cellule (CNRS UMR7654), Ecole Polytechnique, Palaiseau, France; ^2^ Institut Jean-Pierre Bourgin, INRAE-AgroParisTech, University Paris-Saclay, Paris, France; ^3^ INSERM U935-UA09, University Paris-Saclay, Hôpital Paul-Brousse, Paris, France

**Keywords:** vitamin B6, molecular mechanics, force field parametrization, free energy simulation, molecular dynamics

## Abstract

Pyridoxal-5′-phosphate (PLP) is a cofactor in the reactions of over 160 enzymes, several of which are implicated in diseases. Methionine *γ*-lyase (MGL) is of interest as a therapeutic protein for cancer treatment. It binds PLP covalently through a Schiff base linkage and digests methionine, whose depletion is damaging for cancer cells but not normal cells. To improve MGL activity, it is important to understand and engineer its PLP binding. We develop a simulation model for MGL, starting with force field parameters for PLP in four main states: two phosphate protonation states and two tautomeric states, keto or enol for the Schiff base moiety. We used the force field to simulate MGL complexes with each form, and showed that those with a fully-deprotonated PLP phosphate, especially keto, led to the best agreement with MGL structures in the PDB. We then confirmed this result through alchemical free energy simulations that compared the keto and enol forms, confirming a moderate keto preference, and the fully-deprotonated and singly-protonated phosphate forms. Extensive simulations were needed to adequately sample conformational space, and care was needed to extrapolate the protonation free energy to the thermodynamic limit of a macroscopic, dilute protein solution. The computed phosphate pK_*a*_ was 5.7, confirming that the deprotonated, −2 form is predominant. The PLP force field and the simulation methods can be applied to all PLP enzymes and used, as here, to reveal fine details of structure and dynamics in the active site.

## 1 Introduction

Pyridoxal-5′-phosphate, or PLP, is the catalytically-active form of vitamin B6 (Jencks, 1986; Lehninger et al., 2008). It serves as a cofactor in over 160 enzyme reactions (Schneider et al., 2000; Eliot and Kirsch, 2004; Lehninger et al., 2008), participating in the metabolism of molecules with amino groups, such as amino acids and amino sugars. PLP-dependent enzymes catalyze many reactions, for instance decarboxylation, deamination, transamination, or racemization. Approximately 4% of all classified enzyme activities are PLP-dependent (Percudani and Peracchi, 2009). Many PLP enzymes are drug targets, such as GABA aminotransferase and l-DOPA decarboxylase (Kappes et al., 2011). Functional defects in PLP enzymes have been implicated in several diseases (Eliot and Kirsch, 2004).

Other PLP enzymes could be relevant as therapeutic proteins. A strong candidate is methionine-*γ*-lyase, or MGL, which is our focus here. MGL functions as a homo-tetramer, with four identical subunits and four active sites, each located at the interface between two monomers (Sato and Nozaki, 2009). It breaks methionine down into *α*-ketobutyrate, ammonia and methane thiol (Sato and Nozaki, 2009). Cancer cells require large amounts of methionine and cannot survive methionine deprivation (Machover et al., 2019), whereas normal cells are more resistant. Therefore, MGL has been proposed as an anticancer tool, which can digest methionine and reduce the growth of cancer cells (Machover et al., 2019). For therapeutic applications, natural MGLs may not be sufficient, due to immunogenicity and limited stability, activity or specificity. Therefore, engineered variants may be needed (Machover et al., 2019; Lu et al., 2020). Improved understanding is also needed, to guide engineering efforts. Specifically, the enzyme from *Brevibacterium aurantiacum* is a prime candidate for engineering, since it is present in food and has a strong specificity for its Met substrate (Kappes et al., 2011; Machover et al., 2019), but a weaker PLP binding than some other MGL enzymes.

**FIGURE 1:**
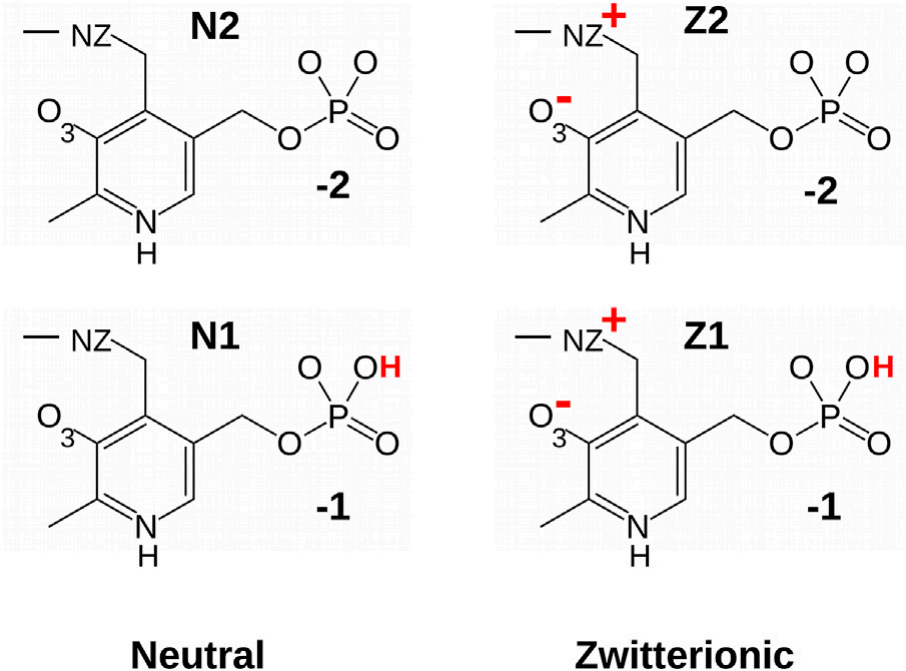
PLP protonation states considered in this work.

Molecular modeling is a powerful tool to study enzyme structure and function. There are specific difficulties for PLP enzymes, including the need for PLP force field parameters (MacKerell et al., 2015) and the existence of several PLP protonation states (Figure 1). The PLP phosphate group can carry a charge of −2 (fully-deprotonated) or −1 (singly-protonated). The pK_*a*_’s of similar phosphate groups in aqueous solution are close enough to 7 so that the protonation state when bound to a protein is not obvious. Although NMR experiments can in principle reveal the phosphate pK_*a*_ under favorable conditions (Limbach et al., 2011; Schnackerz et al., 2011), no measurements have been attempted on MGL. The PLP pyridine ring can also be protonated on its nitrogen, depending on its environment in the protein complex (Lin and Gao, 2010; Limbach et al., 2011; Lin et al., 2011; Richard et al., 2011). Finally, to perform the MGL reaction, PLP should be covalently attached to a lysine side chain, forming a Schiff base linkage (Lehninger et al., 2008; Sato and Nozaki, 2009). The linking atoms exhibit a keto-enol tautomerism, with a proton carried either by the linking nitrogen NZ (zwitterionic, keto form) or by a nearby oxygen O_3_ (neutral, enol form) (Jencks, 1986; Lin and Gao, 2010; Lin et al., 2011). Lin and Gao did a thorough computational study of the keto-enol equilibrium in one PLP enzyme, dopa decarboxylase, using a sophisticated, mixed, quantum/classical model, and found a 1.3 kcal/mol preference for the zwitterionic, keto form (Lin and Gao, 2010; Lin et al., 2011). However, the preference depends on the active site structure and polarity, and may differ in other enzymes, like MGL.

Here, we describe a computational model for MGL enzymes. The model includes several elements that will be of use for PLP enzymes in general. First, we describe the development of force field parameters for four PLP forms, compatible with the Amber protein force field. A simple procedure was used, where charges and geometry were computed from quantum mechanics and most of the other parameters were inferred by analogy to existing groups in the force field. However, special care was needed to describe a dihedral torsion angle that spans the Schiff base linkage. There, quantum calculations did not reproduce observations from the Protein Data Bank (PDB), and so a more empirical procedure was used, where the dihedral parameters were adjusted against the PDB data. Second, we considered the four main PLP protonation states, and modeled each of them through extensive molecular dynamics (MD) simulations of PLP bound to the enzyme from *Pseudomonas putida*, a well-studied bacterial MGL (Sato and Nozaki, 2009; Machover et al., 2019). We refer to it below as ppMGL. To evaluate the simulation models, we compared the structures from MD to those found in 27 PDB structures, including 11 of ppMGL. Overall, the MD models gave good agreement with PDB structures and revealed many details regarding active site structure and fluctuations.

Third, we describe free energy perturbation (FEP) calculations to determine the tautomeric state of the Schiff base and the protonation state of the PLP phosphate when bound to ppMGL. To obtain converged results for the protonation state, the sampling requirements were significant and a detailed analysis of the conformational fluctuations and preferences of the active site was required. Since we compared two states that differed by a net charge (−1 or −2 phosphate), care was also needed to extrapolate the simulation data to the thermodynamic limit of a macroscopic, experimental-like system (Rocklin et al., 2013; Lin et al., 2014; Simonson et al., 2016). The FEP simulations yielded a preference for the keto tautomer by 1.3 ± 1.0 kcal/mol. They gave a phosphate pK_*a*_ of 5.7 and thus a distinct preference for the dinegative form, predicted to represent over 98% of the population at a physiological pH of 7.5.

## 2 Materials and Methods

### 2.1 Molecular Mechanics Force Field

Molecular mechanics parameters for the PLP cofactor were derived in this work, in forms that were covalently bound to lysine (Figure 1). Four protonation states were treated: the phosphate group was singly- or doubly-charged, and the NZ-O_3_ atom pair had a proton either on NZ (zwitterionic, keto form “Z”) or O_3_ (neutral, enol form “N”). The forms are referred to as Z1, Z2, N1, and N2.

Atomic partial charges were calculated using Gaussian09 (Frisch et al., 2009), with the Hartree-Fock method and the 6-31G* basis set (HF/6-31G* method) (Hehre et al., 1987), consistent with the Amber protein force field (Cornell et al., 1995; Maier et al., 2015). The PLP moiety was covalently bound to a fragment of the Lys side chain, truncated after the CD atom (with the CD methylene replaced by a methyl). Charges were adjusted, as usual, to reproduce the electrostatic potential at nearby points selected according to the Merz-Singh-Kollman scheme (Cornell et al., 1995). Atom types for the PLP moiety were assigned by analogy to standard amino acids or nucleotides in the Amber ff14SB force field. Van der Waals parameters were then available automatically. Geometric parameters were taken from the optimized *ab initio* geometries, and force constants were inferred by analogy to existing groups, with one exception. For the Schiff base dihedral angle *χ* = C4-C4′-NZ-CE, a more complex procedure was used, where the distribution sampled in MD simulations was adjusted to match the PDB distribution; see details in Results.

### 2.2 Molecular Dynamics Simulations of MGL

Simulation systems were prepared using Xplor (Brünger, 1992), starting from the ppMGL crystal structure (PDB code 1GC2), which is a homotetramer. The missing loops in 1GC2 were taken from 2O7C, structurally aligned, and minimized by applying harmonic restraints at the junction points, using Xplor. Each model involved a protein tetramer, spherically truncated to eliminate parts far away from the active site. Truncation spheres were centered on the PLP in one specific active site and had radii of 28 Å. Each truncated tetramer was solvated by an octahedral TIP3P water box (Jorgensen et al., 1983). Water molecules having an oxygen less than 2.5 Å from any nonhydrogen protein atom were deleted. A few sodium ions (around 5, depending on the system) were added, to neutralize the total protein charge. The water box had an edge length of 78 Å.

MD simulations were performed with the Amber ff14SB protein force field (Maier et al., 2015) and PLP parameters developed here. Simulations were done with NAMD (Phillips et al., 2005). They used periodic boundary conditions, with a truncated octahedral box and the Particle Mesh Ewald method for long-range electrostatics (Darden et al., 1993). A 12 Å cutoff was applied to van der Waals and real-space electrostatic interactions. Temperature was maintained at 295 K using Langevin dynamics for nonhydrogen atoms with a damping coefficient of 5 ps^−1^. A pressure of 1 bar was maintained by the Langevin piston Nose-Hoover method, with an oscillation period of 200 fs and a damping time of 100 fs (Martyna et al., 1994; Feller et al., 1995).

All simulations followed the same setup procedure. Systems were first minimized for 1,000 conjugate steps, followed by 100 ps of equilibration in the NVT ensemble, then four segments of 500 ps each in the NPT ensemble, with decreasing harmonic restraints in each equilibration segment. For the MD production, only the residues within 5 Å of the edge of the protein truncation sphere were restrained. The harmonic force constants gradually decreased going from the edge inwards, with four concentric shells having values of 2, 1, 0.5 and 0.25 kcal/mol/Å^2^.

### 2.3 PDB Structure Analysis

We analyzed 27 PDB structures that all contained the PLP cofactor, covalently bound to lysine (Lys211 in ppMGL): 11 of ppMGL and 16 of orthologs. Removing symmetry images within each MGL tetramer left 63 independent monomers. The list of structures, including information on ligands and crystallization conditions, is in Supplementary Material, Table S1. Selected structural variables were extracted using Xplor, related to PLP and nearby side chains.

### 2.4 Free Energy Perturbation Simulations

#### 2.4.1 Modeling Deprotonation

**FIGURE 2:**
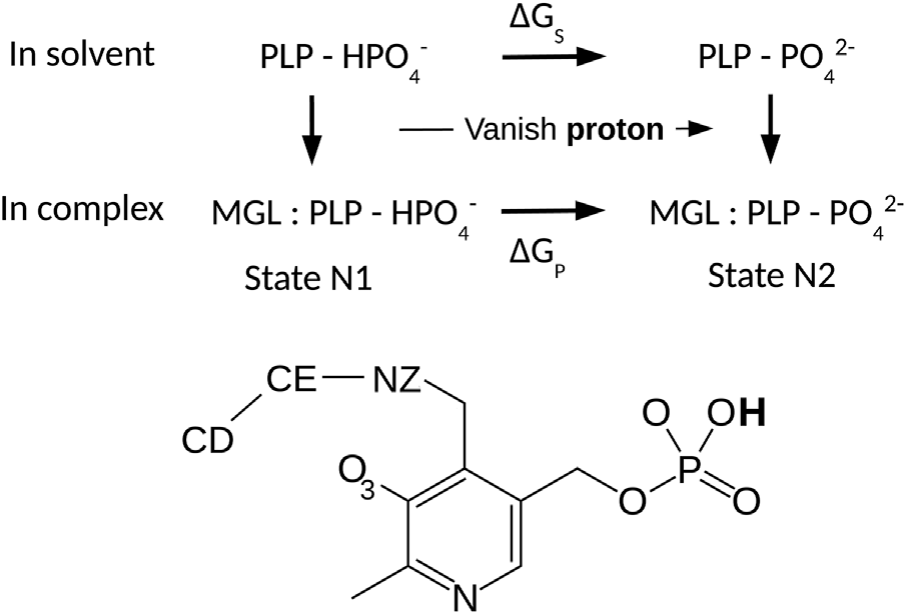
Thermodynamic cycle for phosphate deprotonation. The horizontal legs correspond to deprotonation and have free energies Δ*G*_*S*_ and Δ*G*_*P*_. Vertical legs correspond to PLP:MGL binding. PLP is shown below.

We used an alchemical free energy perturbation method (FEP) to determine the pK_*a*_ value of the PLP phosphate group in the ppMGL-PLP complex, relative to the value for PLP in solution (Limbach et al., 2011). The theory and protocols for pK_*a*_ calculations in proteins by FEP are well-known (Simonson et al., 2004). We considered the thermodynamic cycle in Figure 2. The horizontal legs correspond to deprotonation in the protein and in solution, respectively. The associated free energies are denoted Δ*G*_*P*_ and Δ*G*_*S*_. The pK_*a*_ shift between the protein-PLP complex and PLP in solution is proportional to the difference between the two deprotonation free energies:

$$pK_{a}^{\text{prot}}-pK_{a}^{\text{sol}}=\frac{1}{2.303kT}\left(\Delta G_{P}-\Delta G_{S}\right)$$ (1)

Here, *k* represents the Boltzmann constant and *T* the temperature. The pK_*a*_ of a very similar model compound in solution is 6.3 (Limbach et al., 2011). The free energy changes were computed from a series of MD simulations, where the PLP phosphate changed gradually from protonated to deprotonated. Because the phosphate proton has no van der Waals interactions in the Amber force field, deprotonation could be modeled as a redistribution of atomic charges.

#### 2.4.2 MD Sampling

With the singly-charged, protonated phosphate group, simulations of PLP in the complex (300 ns) and in solution (20 ns) were done first, to obtain equilibrated systems. Selected frames served as starting points for FEP; see Results. We switched the partial charges of PLP from the initial state to the end state through a series of five simulations. We refer to the simulation states by a progress variable *λ*, which varied from 0 to 1. Endpoints and three intermediate values were simulated: *λ* = 0 (singly-protonated), 0.25, 0.5, 0.75, 1 (fully-deprotonated). 40–80 ns were run for each *λ* value, in a series of five simulations referred to as a “run”. In each run, the endpoint (or midpoint) of the simulation at one *λ* value served as the starting point for the next value, e.g., *λ* + 0.25 in the forward direction. The free energy changes for each *λ* interval were estimated both with the Bennett acceptance ratio method (BAR) (Bennett, 1976) and with a thermodynamic integration method (TI) (Simonson et al., 2001). For TI, the free energy derivative *∂G*/*∂λ* at each *λ* value was obtained by a finite-difference approximation (Villa et al., 2018). The derivatives were interpolated using cubic splines and the integral computed analytically, using in-house software. To ensure adequate sampling, several runs were carried out, corresponding to a total simulation time of 1,680 ns.

#### 2.4.3 Extrapolating to the Thermodynamic Limit

Whenever MD is used to study a dilute protein solution, the finite simulation box may introduce artefacts (Kastenholz and Hünenberger, 2006; Lin et al., 2014; Simonson et al., 2016). For an ionic transformation, a neutralizing charge must be introduced, to maintain a neutral simulation box. With PME, this is done through a uniform charge density, or gellium (Lin et al., 2014; Simonson et al., 2016). A side effect is that the mean potential throughout the simulation box is constrained to be zero. The potential is shifted to achieve this, and the shift depends on the box size and composition. In the limit of a large box, the solution and protein systems experience the same shift, so that the effect of the shift cancels when one compares the protein and the model compound. For a finite box size, however, the two shifts will differ. To compare the two systems, we should adjust the protein system in such a way that, far from the protein, in the bulk-like solvent region, the mean potential is the same as in the solution system—namely, zero. To compute the potential throughout the protein simulation box, we used the VMD plugin pmepot (Aksimentiev and Schulten, 2005). The plugin requires a cubic box, and so the protein system (truncated quasi-spherical model) was immersed in a cubic water box whose size was chosen to give the same number of water molecules as the octahedral simulation box. Pmepot computes the potential for each MD snapshot, on a uniform cubic grid that spans the simulation box. Grid points more than 12 Å from any protein atom were considered to be in the bulk-solvent-like region of the box. Averaging over the grid points and the MD snapshots, we obtained a mean electrostatic potential, say Φ. Upshifting it to zero added a contribution to the deprotonation free energy that was simply −1 ×Φ.

A second free energy artefact is the contribution due to the anisotropy of the protein surroundings in the MD setup, and to direct interactions between the proton charge inserted in one box and its replicas in all the other boxes (Lin et al., 2014; Simonson et al., 2016). We refer to it as Δ*G*_self_. A rigorous estimate is obtained by adopting a continuum dielectric view and computing the proton insertion free energy with and without periodic boundary conditions. Unfortunately, a truncated octahedral box is not supported by most continuum electrostatics programs. However, a rougher estimate of Δ*G*_self_ can be obtained by assuming the proton charge is inserted into a truncated octahedral box that has a uniform dielectric composition, with a dielectric constant *ϵ*_box_ averaged over the protein and solvent regions of the box. We used 20 as the value for the protein region, based on MD simulations of six proteins (Simonson, 2003) and we considered that the solvent region was formed by the bulk-like grid points employed above. This led to an average *ϵ*_box_ = 40. The free energy in the large box limit *L* → *∞* was then obtained as

$$\Delta G\left(L\to \infty \right)=\Delta G\left(L\right)+\frac{1}{\epsilon_{\text{box}}}\Delta U_{\text{self}}$$ (2)

where Δ*U*_self_ is the “self” energy, obtained when inserting the proton charge into the periodic array of boxes, computed with NAMD. Thus Δ*G*_self_ = Δ*U*_self_/*ϵ*_box_. We obtained a value of 0.17 kcal/mol. Given the very small magnitude and the uncertainties of the method, we consider this correction can be neglected.

#### 2.4.4 Keto/Enol Comparison

We used the same FEP method to compare the neutral, enol PLP state N2 to the zwitterionic, keto state Z2. We used only two MD windows, corresponding to the two endpoints of the transformation, *λ* = 0 (N2 endpoint) and *λ* = 1 (Z2 end point). However, each endpoint state was sampled extensively, for 1,200 ns each. No experimental N2/Z2 free energy difference was available for PLP in solution. Therefore, we compared the protein system to PLP in the gas phase, for which a quantum mechanical (QM) study was available (Lin and Gao, 2010). For sampling in the gas phase, we used a single, minimized structure for each endpoint. We took care that each of the structures was in accord with the QM structure.

## 3 Results

### 3.1 PLP Force Field Parameterization

The fragments used for charge calculations are shown in Figures 1, 2. PLP in its lysine-bound form was represented by a fragment where the Lys side chain was truncated (after CD) and methylated. To re-establish the correct total charge in the context of a full lysine side chain, the *ab initio* charges obtained for atoms close to the junction were adjusted slightly by hand. Atom types were inferred by analogy to groups in the Amber force field. Geometric parameters were taken from the optimized *ab initio* geometry, with one exception, described next. Final parameters are reported in Supplementary Material, in a format compatible with Xplor (Brünger, 1992) and NAMD (Phillips et al., 2005).

**FIGURE 3:**
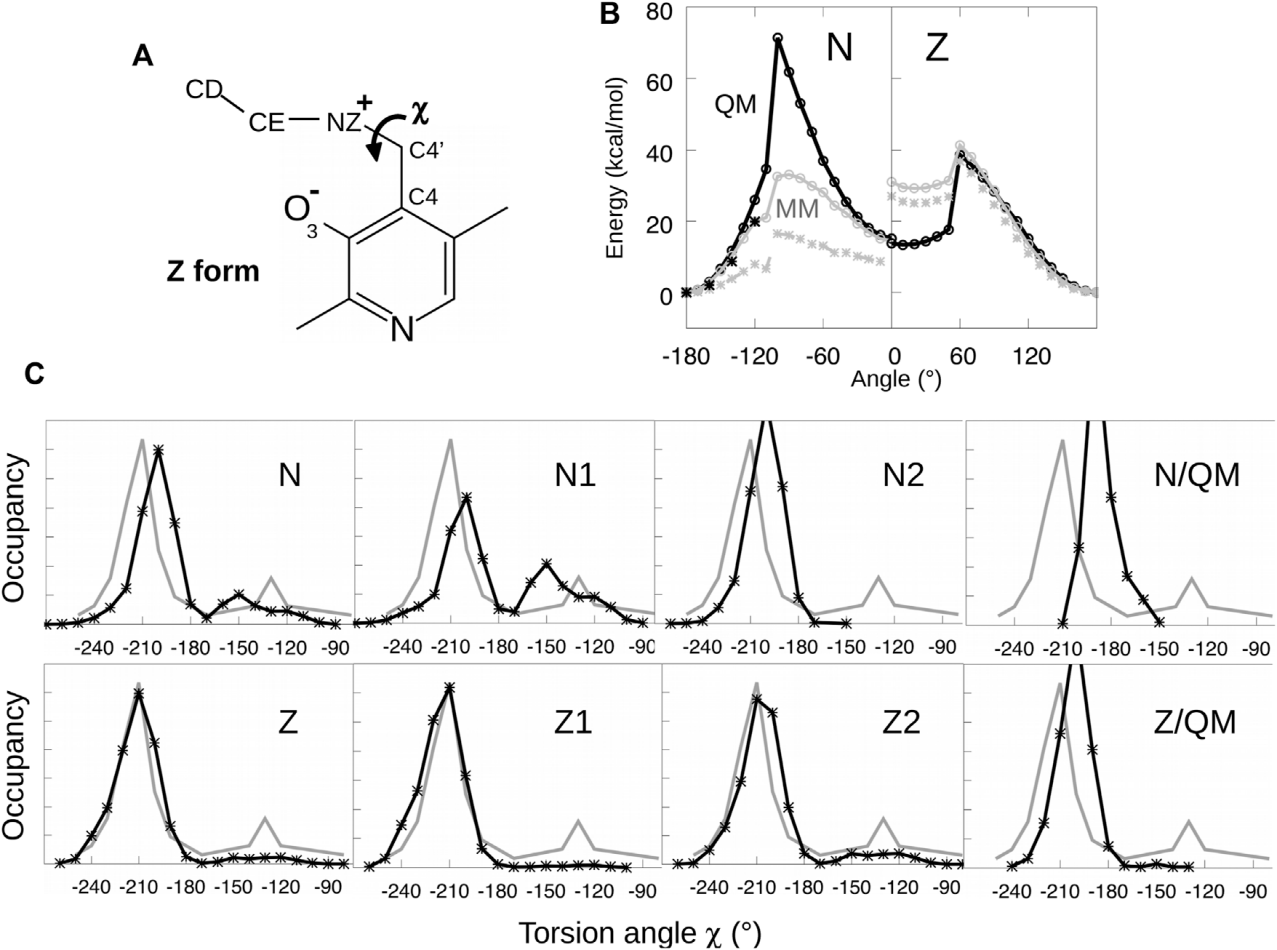
Parametrizing the Schiff base torsion angle *χ* = C4-C4′-NZ-CE. **(A)** Model compounds for N and Z states. **(B)** Energy as a function of *χ* from quantum mechanics (black) and molecular mechanics (gray). Black crosses are from QM calculations at the MP2 level. The MM calculations used parameters tuned to fit the QM curves or the PDB data. **(C)** Probability distribution of *χ* from PDB structures (gray) and MD simulations of the PLP:MGL complex (black).

To optimize parameters for the Schiff base dihedral angle *χ* = C4-C4′-NZ-CE, torsion energy scans were initially performed for both the neutral and zwitterionic forms of the lysine-bound PLP. A model compound was used, shown in Figure 3, having no phosphate group. We restrained the *χ* dihedral angle to a series of values from −180° to 180° and minimized each structure with molecular mechanics, using a restraint force constant of 1,000 kcal/mol/Å^2^ and the Xplor program. The minimized structures served as the starting points for quantum mechanical geometry optimization, with a dihedral constraint and the HF/6-31G* method. Care was taken to maintain the orientation of the groups surrounding the Schiff base linkage, to ensure a smooth variation with *χ* and good consistency between the conformations optimized with molecular mechanics and quantum mechanics. For *χ* angles from −180° to −120°, we re-optimized the structures and calculated the energies with the MP2 method and the 6–311 (1d, 1p) basis set, chosen for consistency with similar torsion energy scans for dihedral parametrization (MacKerell et al., 2015). The molecular mechanics parameters were fitted by comparing the energies from Xplor and Gaussian. Fitted curves are shown in Figure 3.

The fitted torsion parameters were then employed in MD simulations of the enzyme with PLP covalently bound. Despite the accurate fit, the values of *χ* sampled in the simulations did not match the values observed in MGL crystal structures, as shown in Figure 3. Therefore, the parameters were adjusted manually and new simulations were performed. After iterating this procedure several times, the MD simulations reproduced approximately the *χ* distribution inferred from the PDB. The initial and final torsion parameters are reported in Supplementary Material. The parameters for the zwitterionic tautomer (Z1/Z2) reproduced the PDB distribution closely, while the fit was somewhat less good for the N1/N2 forms, despite extensive fitting efforts. The final parameters were then used for all the MD simulations described below.

### 3.2 Analysis of PDB Structures of MGL

Before describing MD simulations of MGL-PLP complexes, we present some aspects of the MGL architecture and the PLP environment. MGL belongs to the cystathionine synthase-like SCOP family, within the PLP-dependent transferase superfamily (Andreeva et al., 2008). In the PDB, 47 MGL crystal structures are found, from seven species. The structures are homotetramers. Each monomer contains an N-terminal domain, a PLP-binding domain and a C-terminal domain. The active sites (four per tetramer) are at the monomer interface within a catalytic dimer.

**FIGURE 4:**
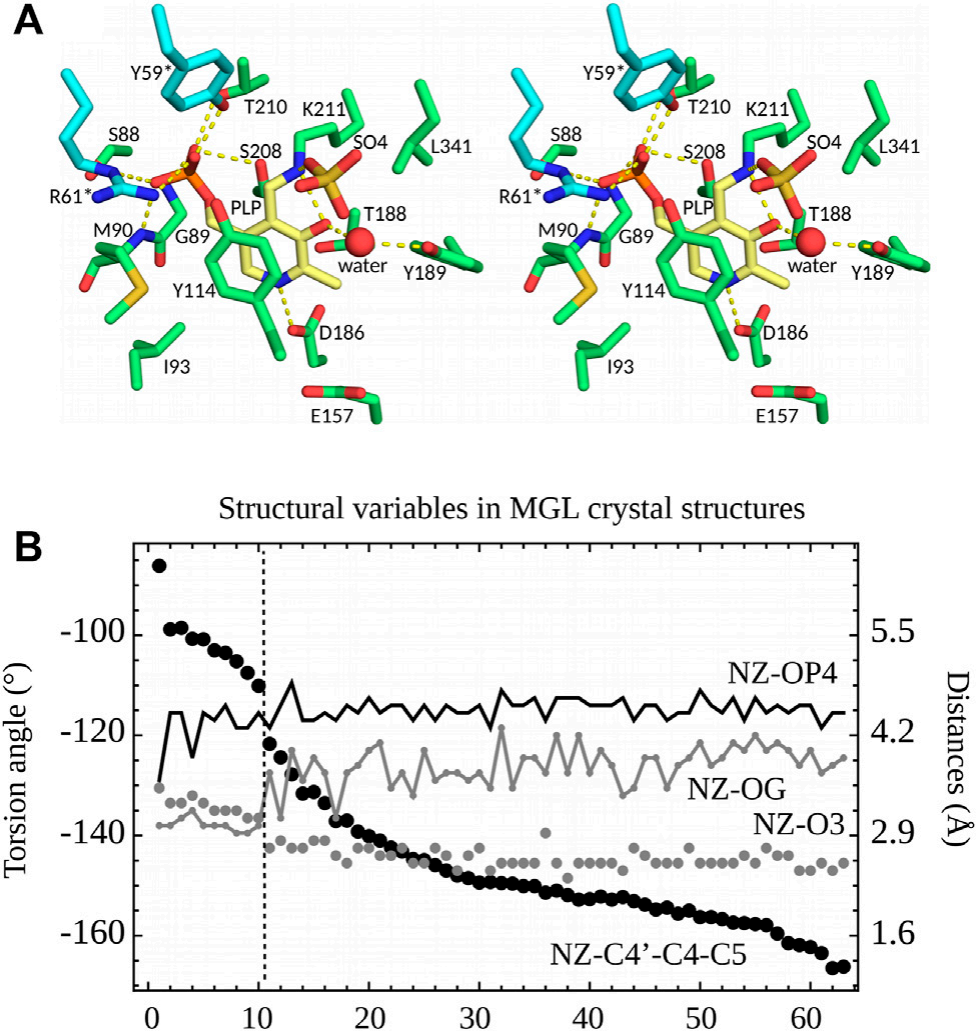
**(A)** 3D stereo view of the ppMGL active site from the 2O7C crystal structure. Selected side chains are shown, a crystal water, and a sulfate ion present in the PDB structure. Selected interactions are highlighted by yellow dashed lines. The PLP pyridine ring is yellow. **(B)** Selected structural variables seen in PDB structures of MGL. Structures are ordered according to the dihedral angle C5-C4-C4′-NZ (black dots). A vertical dashed line separates structures (on the left) where the PLP NZ atom has the B orientation and ones (on the right) with the A orientation.

27 of the PDB structures have PLP bound in the active site through a covalent bond to Lys211 (ppMGL numbering). Protonation states of several active site groups, including PLP, are unknown. The C4′-NZ chemical bond linking PLP to Lys211 can be seen as part of an imine Schiff base, and is expected to have a partial double bond character (Jencks, 1986). Nevertheless, in the PDB structures, the C4-C4′-NZ-CE atoms deviate from a planar geometry, as described in the previous section. Eight of the 27 PDB structures (Table S1) have a crystallization anion directly bound to the PLP NZ atom, which is presumably protonated in these cases (keto form). The anion site is close to the position where the carboxylate of the Met substrate will ultimately bind. All 27 structures were crystallized with either sulfate (0.1–1.8 M) or chloride (50 or 250 mM) anions. The NZ atom has two distinct orientations in the 63 monomers, revealed by the distances between NZ and O3 and OG (Ser208) (ppMGL numbering; Figure 4B). The first orientation, “A”, is towards the anion site. In this orientation, NZ and O3 form a hydrogen bond. “A” occurs in 25 structures, solved with either sulfate or 50 mM chloride crystallization buffers. Six have a visible anion, always sulfate. The second orientation, “B”, has NZ turned inwards, and more buried. It occurs in four structures, including one where A and B are seen in different monomers. All four have chloride crystallization conditions; one (5DX5) has a visible Cl^−^. In two of them (1GC0, 1GC2, both ppMGLs), there is negative density in the Fo—Fc electron density map at the NZ position, which suggests A may also be partially occupied, even though it is absent from the PDB coordinate file. Orientation B gives rise to an NZ-OG (Ser208) hydrogen bond, allowed by both NZ protonation states (since OG can be either donor or acceptor). Though NZ is turned towards the phosphate in the four B structures, it is too far to form a hydrogen bond (Figure 4B). Overall, orientation A is predominant, especially with sulfate or low chloride concentrations.

The PLP aromatic ring stacks upon Y114 (Figure 4A). Its nitrogen atom N1 interacts closely with D186, which indicates (Jencks, 1986) that N1 is protonated. The PLP phosphate forms hydrogen bonds with the S208 and T210 side chains and the G89 and M90 backbone NH groups. In most of the structures, the phosphate interacts with the side chains of Y59* and R61*; the asterisk indicates they are contributed by the other monomer of the catalytic dimer. Out of 63 subunits, 10 have the 52–62 loop disordered (ppMGL numbers); in the other 53, the interactions are present in all but 7 (Y59*) and 5 (R61*), for an occupancy of around 75%. R61* also hydrogen bonds to Y114 in 45 of 63 of the PDB subunits (71%). All these residues are conserved across orthologs. All the interactions are compatible with either a singly-protonated or a fully-deprotonated phosphate. We also expect that either atom O3 or NZ should carry a proton (Figure 1).

### 3.3 Comparing MD Models to PDB Structures

With force field parameters in hand, we proceeded to simulate ppMGL with bound PLP in four distinct protonation states: −1 or −2 phosphate and zwitterionic (keto) or neutral (enol) form of the Schiff base linkage. We refer to these forms as Z1, Z2, N1, N2 (Figure 1). For each form, we ran two MD simulations of 600 ns each, for a total of 4.8 microseconds of simulation. Rms deviations from the starting structures were stable on these timescales, indicating that all the models were reasonably stable, with deviations of 0.5–1 Å for backbone atoms and 1.5–2 Å for side chains. We first describe the interactions of the PLP phosphate group, and compare results for the singly- and doubly-charged forms. Next, we consider other PLP interactions, and compare the results for the neutral and zwitterionic forms.

#### 3.3.1 PLP Phosphate Interactions

**FIGURE 5:**
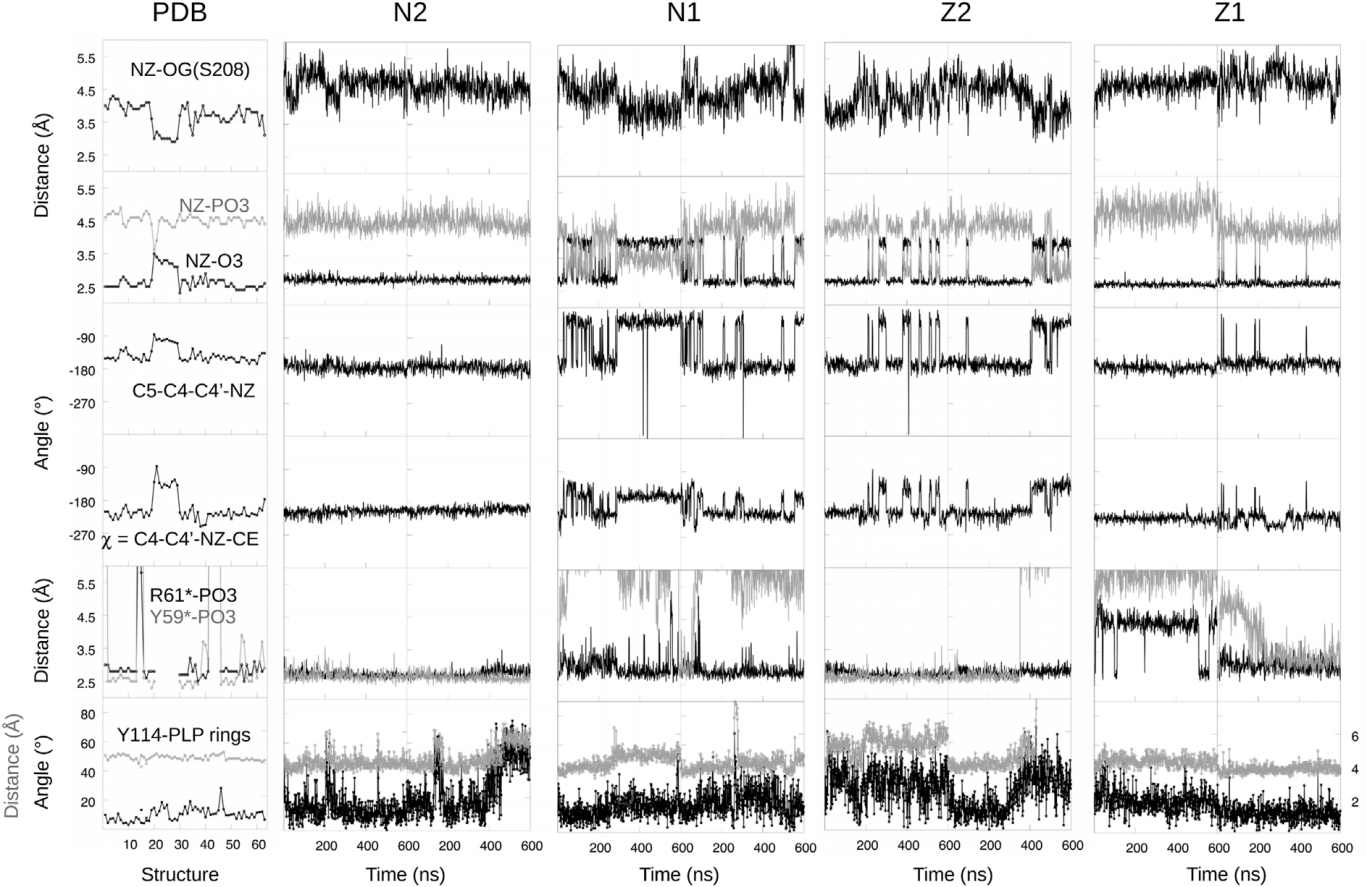
Selected structural variables seen during MD.

In the PDB, the PLP phosphate is about 4.5 Å from the Lys211 NZ atom of the Schiff base linkage. This distance was well maintained in the N2 and Z2 simulations (−2 phosphate). In contrast, with N1, a hydrogen bond was frequently formed, and with Z1, longer distances of around 5.5 Å were frequently seen. In all 63 PDB units, the phosphate hydrogen bonds with the S208 and T210 side chains and with the G89 and M90 backbones. In the simulations, the S208 and backbone interactions were maintained, but the M90 interaction was weaker with a −1 phosphate, with mean distances of 3.05 ± 0.25 Å (N1) and 3.01 ± 0.20 Å (Z1). With a −2 phosphate, the distances were 2.84 ± 0.12 Å (N2) and 2.86 ± 0.12 Å (Z2), in close agreement with the PDB structures, which gave 2.88 ± 0.08 Å. The T210 interaction was almost always maintained with N2 and Z2, but mediated by a water molecule 1/3 of the time with N1 and all the time with Z1. In 3/4 of the PDB subunits, the phosphate hydrogen bonds with Y59* from a neighboring subunit and forms a salt bridge with R61*. In the remaining subunits, either these interactions are broken or the entire 52–62 loop that carries Y59* and R61* is disordered. In the simulations, both interactions were fully maintained with a −2 phosphate (N2 and Z2). With a −1 phosphate, the Y59* interaction was present only 8% of the time, while the R61* salt bridge was maintained in three of four simulations (all but Z1, run 1), albeit with a slightly increased separation (Figure 5). Finally, the PLP phosphate interacted directly with one water molecule in all the simulations. Overall, the largest differences between the two phosphate charge states involved R61* and especially Y59*, with M90 also supporting Z2/N2. The observations above suggest that in the PDB, the −2 phosphate state is predominant. This is consistent with our FEP study of phosphate protonation, below.

#### 3.3.2 Other PLP Interactions

We modeled PLP in both its neutral and zwitterionic states. Several structural variables are plotted in Figure 5. One is the Schiff base dihedral angle *χ* = C4-C4′-NZ-CE. In the PDB, this group is nonplanar, with *χ* values distributed in two peaks, centered at −210° (≡+150°) and −130°, respectively. 3/4 of the subunits are in the first peak. In the MD simulations, only the Z2 model obeys this distribution. N2 and Z1 only visit the first peak, while N1 spends too much time in the second peak (Figure 5). The orientation of the linking NZ atom correlates with *χ*. In most of the PDB structures, NZ has the A orientation, with just a few subunits in the B orientation (away from solvent). This is reflected in the NZ–O3 distance distribution and the C5-C4-C4′-NZ dihedral angle. Among the simulations, N2 and Z1 always occupied A, with small fluctuations, whereas N1 spent too much time in the B orientation and Z2 had a distribution similar to the PDB.

Another important PLP interaction present in all the PDB structures is the Y114 stacking (ppMGL numbering). It was mostly maintained in the MD simulations, but weakened in one Z2 run, where the stacking distance was increased from 4.5 to 5.5 Å most of the time, and the Y114 ring was rotated about 20° away from the well-stacked orientation. In the simulations, a strong interaction seen in the PDB between the pyridine N1 atom and D186 was well-maintained throughout the simulations, except for a portion of N1. Finally, the Z1/Z2 states led to a higher water occupancy near the PLP O3 atom, due to its strongly negative character in these states. With Z2, there was one water within 3 Å throughout; with Z1, there was a water about 1/2 the time. An interaction between the PLP O3 and Y189 also presented differences depending on the PLP state. In the PDB, the mean distance is 4.9 ± 0.3 Å, with no cases of hydrogen bonding. This situation was largely respected in the Z2 simulations, less well with N2, and not very well with N1 and especially Z1.

Overall, the Z2 model gave a slightly weaker Y114 stacking than in the PDB, but better agreement than the other models for the Schiff base angle, the NZ orientation, and the PLP interactions with Y189, D186 and M90.

### 3.4 Free Energy Study of PLP Phosphate Protonation

Phosphate deprotonation was first simulated for the model compound in solution (upper leg, Figure 2). The protonated compound was prepared through 20 ns of MD. The transformation was simulated in five steps, with five distinct *λ* values and 20 ns of MD for each. Each simulation is referred to as a window. The endpoint (or midpoint) of one window served as the starting point for the next. The last frame of the *λ* = 1 window was used to initiate the reverse transformation. Postprocessing was done with BAR. Agreement between the forward and backward runs was excellent, with Δ*G*_*S*_ values of −80.4 and −80.5 kcal/mol, indicating that sampling was adequate. Postprocessing with thermodynamic integration (TI) gave the same results, within 0.1 kcal/mol (Table 1).

**TABLE 1:** FEP results for PLP phospate deprotonation.

*δλ*	Solution	A/A′	B/B′	C/C′	d/d’	-
______________________*δG* (kcal/mol) __________________________________						
0.0 → 0.25	−8.5	−10.7/−14.2	−8.9/−8.9	−7.8/−10.1		-
0.25 → 0.5	−16.3	−16.9/−20.5	−15.7/−15.8	−15.0/−16.1		-
0.5 → 0.75	−23.6	−23.5/−24.0	−21.4/−21.4	−20.5/−21.2	−23.9/−25.0	-
0.75 → 1.0	−32.1	−28.1/−28.1	−28.4/−28.5	−26.7/−26.9	−28.5/−29.1	-
total Δ*G*	−80.5	−79.1/−87.0	−74.5/−74.6	−70.1/−74.3	NA	
runs*λ*	solution	A/A′	B/B′	C/C′	d/d’	Mean
___________________________*∂G*/*∂λ* (kcal/mol) __________________________________________						
0.0	−18.0	−30.5/−39.8	−22.3/−23.3	−18.7/−28.5		−24.6
0.25	−50.3	−54.7/−71.6	−49.3/−50.4	−46.9/−53.7		−51.0
0.5	−79.4	−85.8/−91.3	−74.6/−73.6	−70.5/−73.8	−87.9/−90.2	−81.1
0.75	−109.8	−100.7/−101.3	−98.5/−99.4	−94.5/−96.1	−104.4/−109.3	−104.9
1.0	−147.5	−129.6/−130.5	−132.2/−131.6	−120.4/−120.7	−127.6/−129.5	−129.1
total Δ*G*	−80.4	−79.4/−87.1	−74.6/−75.1	−70.5/−74.7	NA	−78.3
total Δ*G*′	−80.4	-	-	-	-	-81.2

In kcal/mol. Upper part: free energy contributions from BAR, for the different runs and *λ* intervals. Lower part: free energy derivatives and their integrals for the different runs and *λ* points; average over runs on the right. For the solution data, two runs gave almost identical results; only their average is reported.

In proteins, ionic transformations like deprotonation are challenging and require careful sampling. The different FEP runs for the protein complex are schematized in Supplementary Material, Supplementary Figure S1. The complex was first simulated in its N1 state (Figure 5). The MD frame after 50 ns was used to initiate a “forward” FEP run (run A). Each *λ* value was simulated for 40 ns. Each subsequent window was started from the midpoint of the previous window (MD frame after 20 ns). For the final, *λ* = 1 window, the last frame was used to initiate a backward run, done in the same way (run A′). Postprocessing with BAR, the two estimated free energy changes for deprotonation Δ*G*_*P*_ were −79.1 and −87.0 kcal/mol. The large difference indicates insufficient conformational sampling, despite 400 ns of total MD. Per-window contributions from BAR are shown in Table 1, along with free energy derivatives from TI. The A/A’ difference is seen to arise mainly from the *λ* = 0 and 0.25 windows.

**FIGURE 6:**
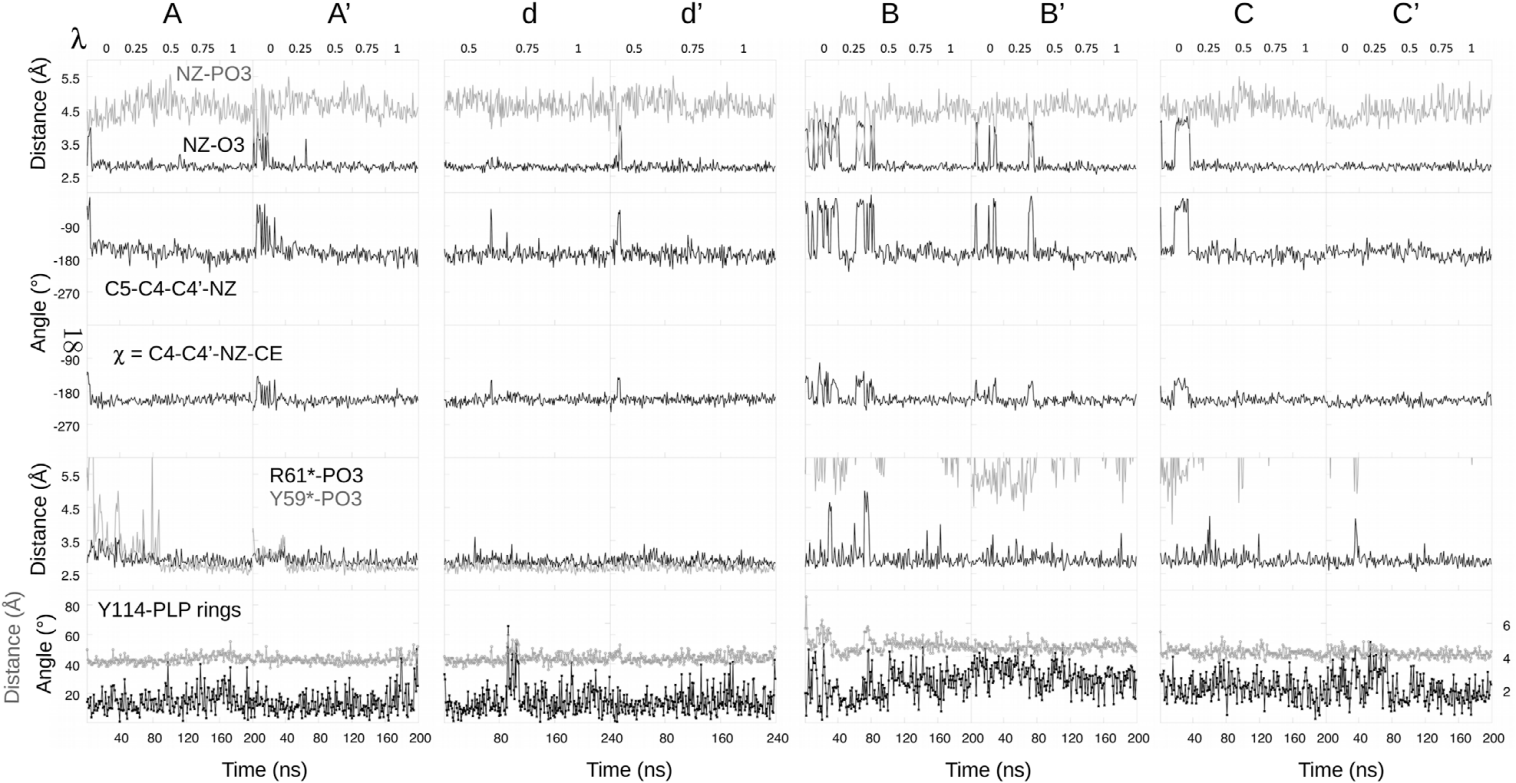
Selected structural variables seen during FEP. Headings above each column indicate free energy runs.

To identify the structures involved, we collected the same structural variables as above. Results are in Figure 6. The main A/A′ difference involves the interaction between the PLP phosphate and Tyr59* from the second subunit. Above (Figure 5), we described a total of 1,200 ns of MD each for the singly- (N1, Z1) and doubly- (N2, Z2) protonated states. The occupancy of the phosphate–Tyr59* interaction was 100% for N2+Z2, presumably due to the −2 phosphate charge. It was around 8% for N1+Z1. In run A, the interaction was present about 1/4 of the time near the N1 endpoint (*λ* = 0, 0.25), then maintained throughout. In the reverse run A′, the interaction was maintained during the entire run. The stronger interaction in the *λ* = 0, 0.25 windows of run A’ led to a more negative Δ*G*_*p*_ value, as expected.

Two additional forward/backward pairs of runs were done in the same way (runs B/B′ and C/C’; Supplementary Figure S1), using state N1 as the starting point, but choosing starting MD frames where the Tyr59*–phosphate was broken (Figure 5). In these runs, each subsequent *λ* window was initiated from the endpoint, not the midpoint of the previous window, thus benefiting from a more thorough equilibration. The structural variables from all these runs are included in Figure 6. In all four runs, the Tyr59*–phosphate interaction never formed, even at the *λ* = 1 endpoints (N2 state). BAR postprocessing gave deprotonation free energies Δ*G*_*P*_ of -74.5/-74.6 kcal/mol for B/B′ and -70.1/-74.3 kcal/mol for C/C’. TI values were very similar, with a mean unsigned TI/BAR difference of 0.3 kcal/mol. Thus, B/B′ and C/C′ were in reasonable agreement with each other and in fair agreement with run A. The deprotonation energies for the first half of the three runs were a bit less favorable than for run A, which is expected, since the Tyr59*–phosphate interaction was present about 1/4 of the time in run A. Run C’ gave somewhat less negative values for all windows.

Finally, another pair of runs was done, which started from the midpoint *λ* = 0.5 value then ran until *λ* = 1 and back (runs d, d’; lowercase names recall that these runs spanned only part of the *λ* range). Window lengths were doubled to 80 ns. The initial frame for run d at *λ* = 0.5 was chosen so that the Tyr59*–phosphate interaction was formed. The interaction was then maintained throughout the two runs. BAR and TI results are included in Table 1.

Overall, based on the 2,400 ns of equilibrium of MD and 1,680 ns of FEP simulations, it appears that for the *λ* ≤ 0.25 range, runs A/B/B’/C/C′ are representative of the most important conformations, with the Tyr59*–phosphate interaction formed 10% of the time (1/4 of run A′). For the range *λ* ≥ 0.75, runs A/A’/d/d’ are representative. For the midpoint window, we assumed we can average over B/B’/C/C’/d/d’. To implement this averaging, we focussed on the TI data, and averaged the free energy derivative values over runs appropriately (A/B/B’/C/C’ for *λ* = 0 and 0.25, and so on). The mean derivatives (Table 1) were then integrated, giving a total deprotonation free energy of −78.3 kcal/mol. This total does not include box size corrections.

Box size corrections were detailed in Methods. Briefly, PME imposes a shift of the electrostatic potential, such that the mean potential throughout the box is zero. For the protein solution, the mean potential far from the protein was obtained by averaging over 100 snaphots from a 10 ns MD run. Around 97,000 grid points out of 372000 were over 12 Å from the protein and considered to represent the bulk solvent region. Averaging over these grid points and MD snapshots, the mean potential was found to be Φ = −2.9 kcal/mol/e. The mean potential in the model compound solution was zero. Upshifting the protein box potential by − Φ brings the potential in the bulk-solvent region to the same level as in the model compound simulation. This shift added a contribution to the deprotonation free energy that was −1 ×Φ = 2.9 kcal/mol. A second free energy correction (Methods) was considered to be negligible.

Thus, we obtained a final value of Δ*G*_*p*_ = −81.2 kcal/mol, and Δ*G*_*p*_ − Δ*G*_*s*_ = −0.8 kcal/mol. The phosphate pK_*a*_ in the protein is given by Eq. 1. The model compound has a solution pK_*a*_ of 6.3 (Limbach et al., 2011), so that the pK_*a*_ in the protein is 5.7. Thus, the PLP phosphate group is predominantly dinegative in MGL. At a physiological pH of 7.5, the dinegative state is predicted to have a population of over 98%.

### 3.5 Free Energy Study of Schiff Base Tautomers

Table 2 reports the N2 → Z2 free energy change in the protein, using a TI method. The free energy derivatives from two separate 600 ns simulations of each state are reported. They were integrated with a trapezoidal method, yielding the free energy change $\Delta G_{N2\to Z2}^{\text{prot}}$ (MM), where MM stands for “molecular mechanics”. In the gas phase, we computed the energy for a single N2 structure and a single Z2 structuree. The gas phase free energy change $\Delta G_{N2\to Z2}^{\text{gas}}$ (MM) is simply the difference. The gas phase value from quantum mechanics $\Delta G_{N2\to Z2}^{\text{gas}}$ (QM) = -0.4 kcal/mol was obtained by Lin and Gao (Lin and Gao, 2010) (B3LYP/6-311G (d,p)//B3LYP/6-311G (d,p) level of theory). Finally, the free difference in the protein was computed as

$$\Delta G_{N2\to Z2}^{\text{prot}}=\Delta G_{N2\to Z2}^{\text{prot}}\left(MM\right)-\Delta G_{N2\to Z2}^{\text{gas}}\left(MM\right)+\Delta G_{N2\to Z2}^{\text{gas}}\left(QM\right)$$ (3)

The final result was $\Delta G_{N2\to Z2}^{\text{prot}}$ = −1.3 ± 1.0 kcal/mol. The uncertainty was estimated by taking results for the two halves of the protein MD segment for each endpoint. The FEP calculations thus indicate a moderate preference for the zwitterionic, keto state Z2. This is consistent with our MD/PDB comparison, above. The preference is, coincidentally, the same as the value obtained by Lin and Gao for another PLP enzyme (Lin and Gao, 2010).

**TABLE 2:** FEP for PLP N2/Z2 equilibrium.

*λ*	___________*∂G*/*∂λ* (kcal/mol) __________	
	vacuum	protein
0.0		−32.9/−34.7^*a*^
1.0		−54.9/−57.1^*a*^
Δ*G* (MM)	−44.0	−44.9 ± 1.0
Δ*G* (N2 → Z2)	−0.4^*b*^	−1.3 ± 1.0^*c*^

Free energy derivatives and integrals. ^*a*^From two 600 ns simulations. ^*b*^From quantum mechanics (Lin and Gao, 2010). ^*c*^From Eq. 3.

## 4 Discussion

To understand and engineer PLP-dependent enzymes, it is important to identify the important tautomeric and protonation states in the active site, especially those of PLP. Our strategy was to model four main states and identify those that best reproduced PDB data. This is not quite straightforward, since there could be several states populated in the PDB, and the populations could differ slightly from the solution populations, due to crystal ions or reduced solvent content, for example. In addition, the simulations used the PDB data as input to parameterize the Schiff base dihedral angle *χ*. In the parametrization, both the neutral and zwitterionic tautomers were assumed to be well-represented by the *χ* distribution in the PDB.

Fortunately, the assumption that PLP states can be distinguished by comparing to the PDB was supported by the data. There was a clear difference between the states with a −1 and −2 phosphate charge, with the −2 states giving a distinctly better match to the PDB. This strongly suggests that the −2 state is predominant in the PDB. In contrast, while the Z2 state gave better agreement than N2, the difference was less marked, and the two states sampled quite similar conformations. From the physical-chemical point of view, the N and Z tautomers do not differ as strongly as the −1 and −2 phosphate states. It may be that the PDB contains a mixture of the two tautomers. The predominance of Z2, compared to N2, supports the parametrization procedure for the Z tautomers. For the N tautomers, the parametrization procedure did not reproduce the PDB distribution as well, despite many fitting attempts. However, if N is a minor state, a slightly poorer fit is probably acceptable. The preference for the zwitterionic, keto tautomer matches that observed by Lin et al. for l-dopa decarboxylase (Lin and Gao, 2010). For the best, Z2 state, agreement between the MD and PDB structures was very good.

We used alchemical free energy perturbation simulations to further test the zwitterionic, keto preference and the predominant phosphate state. FEP yielded a moderate preference for Z2 over N2, with a free energy difference of 1.3 ± 1.0 kcal/mol. Phosphate protonation was carried out for the neutral, enol tautomer (N1 vs N2), but we expect the conclusions are also valid for the zwitterionic, keto tautomer (Z1 vs Z2). Indeed, the NZ and O3 atoms do not directly interact with the PLP phosphate; the position of their shared proton should not couple too strongly to the phosphate charge, and the conformations sampled with the N and Z states were not very different. To obtain converged protonation free energies, we employed almost 1.7 microseconds of MD, about three times as much as in a recent study of some other ionic mutations from our group (Villa et al., 2018). In addition, averaging over the different free energy runs was informed by the 4.8 microseconds of sampling performed for the main PLP states, prior to FEP. More sophisticated sampling methods, such as smart, adaptive umbrella sampling (Zheng et al., 2008; Lu et al., 2016), should be explored but are beyond the scope of the present work. We evaluated a free energy contribution arising from the PME simulation model, which applies a potential shift to the protein and solution simulation systems. The magnitude of this contribution was significant, underlying how important it is to correct for this simulation artefact. Overall, we obtained a computed phosphate pK_*a*_ of 5.7, which corresponds to a strong preponderance of the −2 form. Notice that in addition to the need for sufficient conformational sampling, FEP may also be limited by force field accuracy, and by the use of a fixed charge model that does not include explicit electronic polarization. However, polarizable simulations are complex and expensive, and beyond the scope of this work. For a phosphate site that is not too deeply buried, as here, we expect that the nonpolarizable force field gives the correct trend (Villa et al., 2018). We also expect that the phosphate pK_*a*_ will be similar in other MGLs, including those in the PDB.

MD is a powerful tool to understand and engineer enzymes. The PLP force field developed here can be applied to other PLP enzymes. The simulation methodology, including the technical details of our FEP approach, can also be applied. In addition to evaluating the four main PLP states, the simulations revealed many details that were not evident in the PDB structures. These include subtle differences between the PLP states, information on the water molecules in the active site, and the extent of disorder for active site groups like Y59*, R61*, and Y114. All these data will help to inform future efforts to engineer a therapeutic MGL, such as the MGL from *Brevibacterium aurantiacum*. The simulation model will be directly applicable to this and other homologs, to other states of the protein, such as MGL with bound substrate, and to engineered variants that are thought to have improved properties.

## Data Availability

The original contributions presented in the study are included in the article/Supplementary Material, further inquiries can be directed to the corresponding author.
