# Supplementary material for: A Computational Model for the PLP-Dependent Enzyme Methionine γ-Lyase
Source: Front Mol Biosci. 2022 Apr 26;9:886358. doi: 10.3389/fmolb.2022.886358 (PMC9087591; doi:10.3389/fmolb.2022.886358)
Supplement: Supplementary file 1 [file DataSheet1.pdf]

Supplementary Material

A computational model for the PLP-dependent enzyme methionine γ-lyase

Xingyu Chen, Pierre Briozzo, David Machover & Thomas Simonson

Table S1 lists MGL crystal structures with crystallization conditions.
Figure S1 schematizes the FEP runsperformed for PLP phosphate deprotonation.
Table S2 lists the PLP atoms with their atom types and charges.
Topology and parameters for PLP are given next as a text file.
The file structures.zip contains structures, velocities and PSF files for the solvated protein with all four PLP protonation states, from snapshots after 600 ns of MD.

Table S1. MGL crystal structures from the PDB

Species	PDB	Resolution (Å)	Crystallization condition	Mutations	PLP bound residues	Co-bound molecules near PLP	NZ orientation (Schiff-base Lys)
Pseudomonas putida	1GC0	1.7	250 mM NaCl	None	PLP-LYS211	None	B
	1GC2	2	250 mM NaCl	None	PLP-LYS211	None	B
	1PG8	2.68	N/A *	None	PLP-LYS211	SO4	A
	1UKJ	1.8	N/A *	None	PLP-LYS211	SO4	A
	2O7C	1.7	100 mM Ammonium sulfate	None	PLP-LYS211	SO4	A
	3VK2	2.3	250 mM Ammonium sulfate	C116H	PLP-LYS211	SO4	A
	3VK3	2.1	250 mM Ammonium sulfate	C116H	PLP-LYS211	MET	A
	3VK4	2.61	250 mM Ammonium sulfate	C116H	PLP-LYS211	HCY	A
	5X2V	2.4	250 mM Ammonium sulfate	None	PLP-LYS211	None	A
	5X2W	2.7	250 mM Ammonium sulfate	None	PLP-MET	None	N/A
	5X2X	2	250 mM Ammonium sulfate	None	PLP-HAL	H_2S	N/A
	5X2Y	1.79	250 mM Ammonium sulfate	C116H	PLP-LYS211	None	A
	5X2Z	1.8	250 mM Ammonium sulfate	C116H	PLP-MET	None	N/A
	5X30	1.7	250 mM Ammonium sulfate	C116H	A: PLP-LYS211	HCY	A
					B: PLP-HAL	H2S	N/A
					C: PLP-LYS211	HCY	A
					D: PLP-HCY	None	N/A

Clostridium sporogenes	5DX5	2.37	50 mM TRISHCL (PH=8.5)	None	PLP-LYS212	Cl	B
Citrobacter freundii	5D5S	1.7	50 mM TRISHCL (PH=8.5)	S339A	PLP-LYS210	None	B
	5E4Z	2.27	50 mM TRISHCL (PH=8.5)	C115A	PLP-LYS210	None	A
	5K30	1.59	50 mM TRISHCL (PH=8.5)	Sulfoxide CYS	PLP-LYS210	None	A
	5M3Z	1.45	50 ML TRIS-HCL	C115H	PLP-LYS210	NLE	A
					PLP-NLE	None	N/A
	6EGR	1.45	50 mM TRIS-HCL	V358Y	PLP-LYS210	None	A
	6S0C	1.46	50 mM TRIS-HCL	Sulfoxide CYS	PLP-LYS210	None	A
	1Y4I	1.9	100 mM Ammonium sulfate	None	PLP-LYS210	None	A
	2RFV	1.35	50 mM TRIS-HCL	None	PLP-LYS210	None	A
	3JW9	1.8	50 mM TRIS-HCL	None	PLP-LYS210	S-ethyl-L-cysteine (ECX)	A
	3JWA	1.45	50 mM TRIS-HCL	None	PLP-LYS210	1-AMINO-3-METHYLSULFANYL-PROPYL)-PHOSPHINIC ACID	A
	3JWB	1.63	50 mM TRIS-HCL	None	PLP-LYS210	norleucine (NLE)	A
	3MKJ	1.65	200 mM Ammonium sulfate; 50 mM TRIS-HCL	Modified CYS	PZP (pyridoximine-5'-phosphate)	None	N/A
	4HF8	2.45	50 mM TRIS-HCL	modified CYS	PLP-GLY	None	N/A
	4MKJ	1.85	50 mM TRIS-HCL	C115A; modified by allicine	PLP-LYS210	Na (close to N1 of PLP)	A
	4MKK	1.45	50 mM TRIS-HCL	C115A; modified by allicine	PLP-LYS210	K	A
	4OMA	1.6	50 mM TRIS-HCL	None	PLP-LCS (L-cycloserine)	None	N/A
	4P7Y	1.96	50 MM TRIS-HCL	Y58F	PLP-LYS210	None	A
Entamoeba histolytica	3ACZ	1.97	1.8 M Ammonium sulfate; PH 6.6	None	PLP-LYS205	SO4	A
	3AEJ	2.59	1.8 M Ammonium sulfate; PH 6.6	None	A, B, D: PLP-MET (AA5)	None	N/A
					C: PLP-LYS205	MET	A
	3AEL	2	1.8 M Ammonium sulfate; PH 6.6	None	A: PLP-HAL	CH4S	N/A
					B, C, D: PLP-MET (2ML)	None	N/A
	3AEM	2.2	1.8 M Ammonium sulfate; PH 6.6	None	A, B, D: PLP-MET (2ML)	None	N/A
					C: PLP-LYS205	MET	A

	3AEN	2	1.8 M Ammonium sulfate; PH 6.6	None	A: PLP-LYS205	MET	A
					B, D: PLP-HAL	None	N/A
					C: PLP-LYS205	SO4	A
	3AEO	2.15	1.8 M Ammonium sulfate; PH 6.6	None	PLP-MET (3ML)	None	N/A
	3AEP	2.28	1.8 M Ammonium sulfate; PH 6.6	None	A, C: PLP-HAL	CH4S	N/A
					B, D: PLP-HAL	None	N/A
Trichomonas vaginalis 1	1E5E	2.18	3.2 M Ammonium sulfate; 0.2 M LISO4	None	PLP-NORVALINE	None	N/A
	1E5F	2.18	3.2 M Ammonium sulfate; 0.2 M LISO4	None	PLP	SO4	N/A
Trichomonas vaginalis 2	1PFF	2.5	100 MM HEPES/NAOH (PH 7.5), 100 MM MGCL2	None	apo	None	N/A
Fusobacterium nucleatum	7BQW	2.5	0.2 M Sodium formate	None	apo	None	N/A
Micromonospora echinospora	4Q31	2.1	PH 6.5	None	PLP-LYS	2-(N-MORPHOLINO)-ETHANESULFONIC ACID	N/A
	4U1T	2	0.2 M Sodium formate	None	PLP-LYS	SO4	A
	4U2H	1.85	0.2 M Sodium formate	None	apo	SO4	N/A
	4XQ2	2.1	PH 6.5	D7G	A, C, E, G: PLP-LYS197	MES	A
					B, D, F, H: PLP-LYS197	MES; GOL	A

Figure S1 Schematic view of FEP runs for PLP phosphate deprotonation.

Runs are named A, A', B, B',C, C', d, d'. Coupling constant λ values are marked by a dot; MD for each window runs vertically. Dashed gray lines indicate the use of a snapshot from one window to initiate the next window. Starting structures or "frames" are indicated for each run.

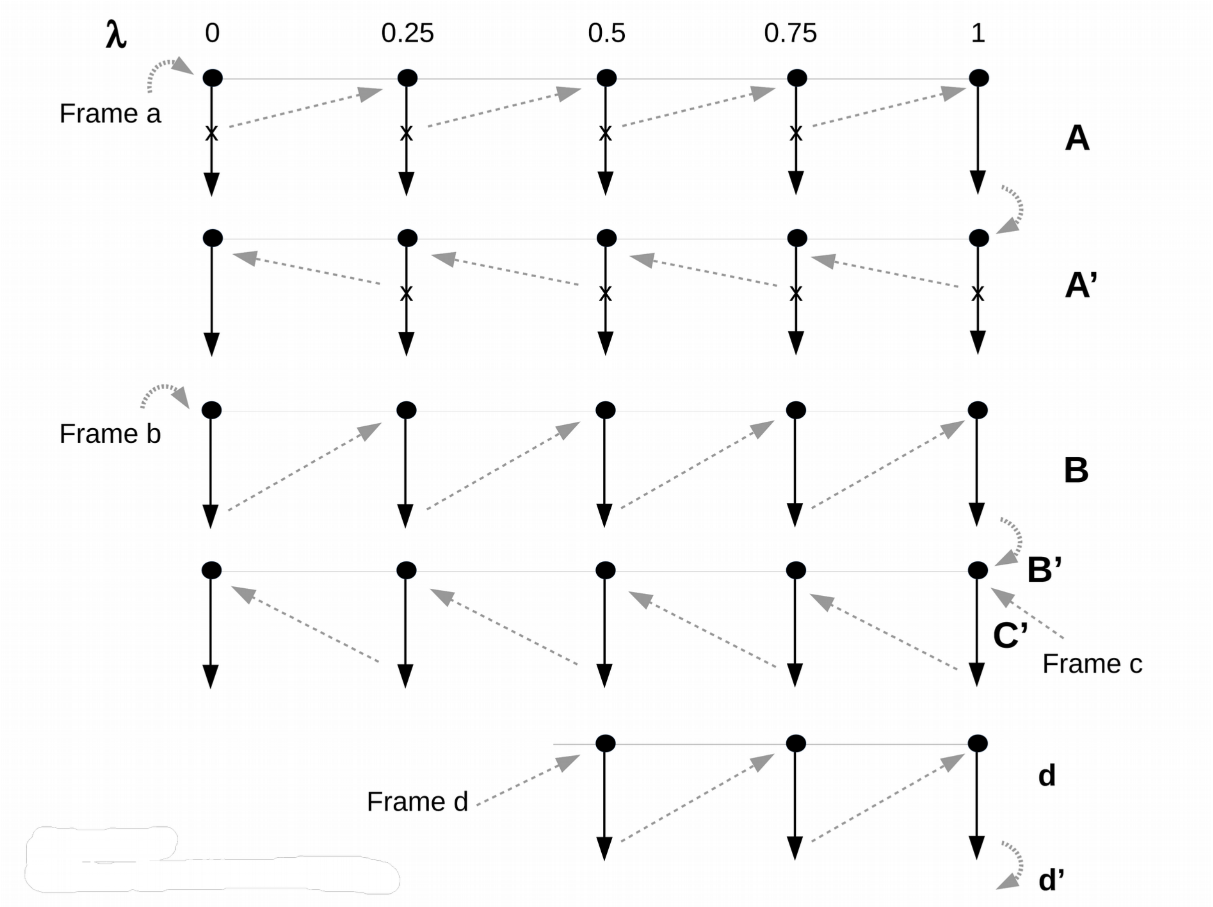

Table S2. Atom types and partial charges of PLP for each form

		N2	N1	Z2	Z1
Atom name	Atom type	Partial charges			
N	N	-0.4157	-0.4157	-0.3479	-0.3479
H	H	0.2719	0.2719	0.2747	0.2747
CA	CX	-0.07206	-0.07206	-0.24	-0.24
HA	H1	0.0994	0.0994	0.1426	0.1426
CB	C8	-0.04845	-0.04845	-0.0094	-0.0094
HB2	HC	0.034	0.034	0.0362	0.0362
HB3	HC	0.034	0.034	0.0362	0.0362
CG	C8	0.06612	0.06612	0.0187	0.0187
HG2	HC	0.01041	0.01041	0.0103	0.0103
HG3	HC	0.01041	0.01041	0.0103	0.0103
CD	C8	-0.03183	-0.03183	-0.2746	-0.2746
HD2	HC	0.0207	0.0207	0.0565	0.0565
HD3	HC	0.0207	0.0207	0.0565	0.0565
CE	C8	0.0668	0.0668	0.4508	0.4508
HE2	HP	0.034	0.034	-0.0297	-0.0297
HE3	HP	0.034	0.034	-0.0297	-0.0297
NZ	NA/N2	-0.3868(N2)	-0.3868(N2)	-0.5364(NA)	-0.5364(NA)
HZ1	H	------	------	0.379	0.379
C	C	0.5973	0.5973	0.7341	0.7341
O	O	-0.5679	-0.5679	-0.5894	-0.5894
P	P	1.3616	1.3116	1.3618	1.3116
C5'	CT	0.2992	0.3116	0.2992	0.3116
C4'	CD	0.1278	0.1278	0.4311	0.4311
C2'	CT	-0.5039	-0.5039	-0.5243	-0.5243
N1	NC	-0.3561	-0.3561	-0.2325	-0.2325
C2	CA	0.2801	0.2801	0.274	0.274
C3	CA	0.197	0.197	0.4435	0.4435
O3	OH	-0.5727	-0.5727	-0.6723	-0.6723
C4	CA	0.1128	0.1128	-0.2913	-0.2913
C5	CA	-0.0523	-0.0523	0.106	0.106
C6	CA	-0.0903	-0.0903	-0.3155	-0.3155
OP1	O2/OH	-0.8876(O2)	-0.6689(OH)	-0.8876(O2)	-0.6689(OH)
OP2	O2	-0.8876	-0.7593	-0.8876	-0.7593
OP3	O2	-0.8876	-0.7593	-0.8876	-0.7593
OP4	OS	-0.5606	-0.5175	-0.5606	-0.5175
H13	H1	0.0094	0.0457	-0.0076	0.0288
H14	H1	0.0094	0.0457	-0.0076	0.0288

H15	H4	0.1971	0.1971	0.1664	0.1664
H16	HC	0.1624	0.1624	0.1596	0.1596
H17	HC	0.1624	0.1624	0.1596	0.1596
H18	HC	0.1624	0.1624	0.1596	0.1596
H19	HO	------	0.4466	------	0.4466
H20	HA	0.1741	0.1741	0.1965	0.1965
H21	HO	0.3793	0.3793	------	------
H22	H	0.3867	0.3867	0.3684	0.3684
Net charge		-1	0	-1	0

Topology and parameters files for PLP

```
! topology file for PLP; X Chen & T Simonson, 2022
! this file: PLP.rtf
! to use: comment out all but one PLP form (eg, below, only N2 is uncommented)


SET ECHO=FALSE END

AUTOGEN ANGL=TRUE DIHE=TRUE END

! topology of PLP in N2 form
!-------------------------------

RESI PLP
GROUP
ATOM P    TYPE=P    CHARGE= 1.361600 END !                  H18
ATOM C5'  TYPE=CT   CHARGE= 0.299200 END !                   |
ATOM C5'  TYPE=CT   CHARGE= 0.299200 END !              H16-C2'-H17
ATOM C4'  TYPE=CD   CHARGE= 0.127800 END !      H21-O3    /
ATOM C2'  TYPE=CT   CHARGE=-0.503900 END !                \  /
ATOM N1   TYPE=NC   CHARGE=-0.356100 END !                C3--C2
ATOM C2   TYPE=CA   CHARGE= 0.280100 END !                   //    \\
ATOM C3   TYPE=CA   CHARGE= 0.197000 END   ---NZ= C4'--C4      N1--H22
ATOM O3   TYPE=OH   CHARGE=-0.572700 END !      |            \ __  /
ATOM C4   TYPE=CA   CHARGE= 0.112800 END !      H15          C5   C6
ATOM C5   TYPE=CA   CHARGE=-0.052300 END !                    /       \
ATOM C6   TYPE=CA   CHARGE=-0.090300 END !            H13-C5'      H20
ATOM OP1  TYPE=O2   CHARGE=-0.887600 END !    (2-)         /  \
ATOM OP2  TYPE=O2   CHARGE=-0.887600 END !   OP3=P--OP4 H14
ATOM OP3  TYPE=O2   CHARGE=-0.887600 END !           /\
ATOM OP4  TYPE=OS   CHARGE=-0.560600 END !     OP2 OP1
ATOM H13  TYPE=H1   CHARGE= 0.009400 END
ATOM H14  TYPE=H1   CHARGE= 0.009400 END
ATOM H15  TYPE=H4   CHARGE= 0.197100 END
ATOM H16  TYPE=HC   CHARGE= 0.162400 END
ATOM H17  TYPE=HC   CHARGE= 0.162400 END
ATOM H18  TYPE=HC   CHARGE= 0.162400 END
ATOM H20  TYPE=HA   CHARGE= 0.174100 END
ATOM H21  TYPE=HO   CHARGE= 0.379300 END
ATOM H22  TYPE=H    CHARGE= 0.386700 END
BOND P     OP1
BOND P     OP2
BOND P     OP3
BOND P     OP4
```

```
BOND C5'   C5
BOND C5'   OP4
BOND C5'   H13
BOND C5'   H14
BOND C4'   C4
BOND C4'   H15
BOND C2'   C2
BOND C2'   H16
BOND C2'   H17
BOND C2'   H18
BOND N1    C2
BOND N1    C6
BOND N1    H22
BOND C2    C3
BOND C3    O3
BOND C3    C4
BOND C4    C5
BOND C5    C6
BOND C6    H20
BOND O3    H21
IMPR  C3  C5  C4  C4'
IMPR  C4  C6  C5  C5'
IMPR  C5  N1  C6  H20
IMPR  C6  C2  N1  H22
IMPR  N1  C3  C2  C2'
IMPR  C2  C4  C3  O3
DONO H22  N1
DONO H21  O3
ACCE OP1  P
ACCE OP2  P
ACCE OP3  P
END

PRES COVA
DELE ATOM 1HZ2  END
DELE ATOM 1HZ3  END
MODI ATOM 1CD   TYPE=C8   CHARGE=-0.031830 END
MODI ATOM 1HD2  TYPE=HC   CHARGE= 0.020700 END
MODI ATOM 1HD3  TYPE=HC   CHARGE= 0.020700 END
MODI ATOM 1CE   TYPE=C8   CHARGE= 0.066800 END
MODI ATOM 1HE2  TYPE=HP   CHARGE= 0.034000 END
MODI ATOM 1HE3  TYPE=HP   CHARGE= 0.034000 END
MODI ATOM 1NZ   TYPE=N2   CHARGE=-0.386800 END
ADD  BOND 1NZ   2C4'
ADD  ANGL 1CE   1NZ   2C4'
```

```
ADD  ANGL 1NZ   2C4'  2C4
ADD  ANGL 1NZ   2C4'  2H15
ADD  DIHE 2C5   2C4   2C4'  1NZ
ADD  DIHE 2C3   2C4   2C4'  1NZ
ADD  DIHE 2C4   2C4'  1NZ   1CE  MULT 2
ADD  DIHE 2H15  2C4'  1NZ   1CE
ADD  DIHE 2C4'  1NZ   1CE   1HE2
ADD  DIHE 2C4'  1NZ   1CE   1HE3
ADD  DIHE 2C4'  1NZ   1CE   1CD
ADD  IMPR 2C4   1NZ   2C4'  2H15
END


{  the pair of brackets opened here  inactives all lines in between

! topology of PLP in N1 form
! ---------------------------------

RESI PLP
GROUP
ATOM P    TYPE=P    CHARGE= 1.311600 END!                  H18
ATOM C5'  TYPE=CT   CHARGE= 0.311600 END!                   |
ATOM C4'  TYPE=CD   CHARGE= 0.127800 END!              H16-C2'-H17
ATOM C2'  TYPE=CT   CHARGE=-0.503900 END!         H21-O3    /
ATOM N1   TYPE=NC   CHARGE=-0.356100 END!             \   /
ATOM C2   TYPE=CA   CHARGE= 0.280100 END!             C3--C2
ATOM C3   TYPE=CA   CHARGE= 0.197000 END!            //    \\
ATOM O3   TYPE=OH   CHARGE=-0.572700 END! ---NZ= C4'--C4       N1--H22
ATOM C4   TYPE=CA   CHARGE= 0.112800 END!        |     \  __  /
ATOM C5   TYPE=CA   CHARGE=-0.052300 END!        H15    C5--C6
ATOM C6   TYPE=CA   CHARGE=-0.090300 END!             /    \
ATOM OP1  TYPE=OH   CHARGE=-0.668900 END!         H13-C5'   H20
ATOM OP2  TYPE=O2   CHARGE=-0.759300 END!      (-)    /  \
ATOM OP3  TYPE=O2   CHARGE=-0.759300 END!   OP3=P--OP4    H14
ATOM OP4  TYPE=OS   CHARGE=-0.517500 END!      /\
ATOM H13  TYPE=H1   CHARGE= 0.045700 END!    OP2 OP1
ATOM H14  TYPE=H1   CHARGE= 0.045700 END!         |
ATOM H15  TYPE=H4   CHARGE= 0.197100 END!        H19
ATOM H16  TYPE=HC   CHARGE= 0.162400 END
ATOM H17  TYPE=HC   CHARGE= 0.162400 END
ATOM H18  TYPE=HC   CHARGE= 0.162400 END
ATOM H19  TYPE=HO   CHARGE= 0.446600 END
ATOM H20  TYPE=HA   CHARGE= 0.174100 END
ATOM H21  TYPE=HO   CHARGE= 0.379300 END
ATOM H22  TYPE=H    CHARGE= 0.386700 END
```

```
BOND P     OP1
BOND P     OP2
BOND P     OP3
BOND P     OP4
BOND C5'   C5
BOND C5'   OP4
BOND C5'   H13
BOND C5'   H14
BOND C4'   C4
BOND C4'   H15
BOND C2'   C2
BOND C2'   H16
BOND C2'   H17
BOND C2'   H18
BOND N1    C2
BOND N1    C6
BOND N1    H22
BOND C2    C3
BOND C3    O3
BOND C3    C4
BOND C4    C5
BOND C5    C6
BOND C6    H20
BOND O3    H21
BOND OP1   H19
IMPR  C3  C5  C4  C4'
IMPR  C4  C6  C5  C5'
IMPR  C5  N1  C6  H20
IMPR  C6  C2  N1  H22
IMPR  N1  C3  C2  C2'
IMPR  C2  C4  C3  O3
DONO H22  N1
DONO H21  O3
DONO H19  OP1
ACCE OP2  P
ACCE OP3  P
END

PRES COVA
DELE ATOM 1HZ2  END
DELE ATOM 1HZ3  END
MODI ATOM 1CD   TYPE=C8   CHARGE=-0.031830 END
MODI ATOM 1HD2  TYPE=HC   CHARGE= 0.020700 END
MODI ATOM 1HD3  TYPE=HC   CHARGE= 0.020700 END
MODI ATOM 1CE   TYPE=C8   CHARGE= 0.066800 END
```

```
MODI ATOM 1HE2  TYPE=HP   CHARGE= 0.034000 END
MODI ATOM 1HE3  TYPE=HP   CHARGE= 0.034000 END
MODI ATOM 1NZ   TYPE=N2   CHARGE=-0.386800 END
ADD  BOND 1NZ   2C4'
ADD  ANGL 1CE   1NZ   2C4'
ADD  ANGL 1NZ   2C4'  2C4
ADD  ANGL 1NZ   2C4'  2H15
ADD  DIHE 2C5   2C4   2C4'  1NZ
ADD  DIHE 2C3   2C4   2C4'  1NZ
ADD  DIHE 2C4   2C4'  1NZ   1CE  MULT 2
ADD  DIHE 2H15  2C4'  1NZ   1CE
ADD  DIHE 2C4'  1NZ   1CE   1HE2
ADD  DIHE 2C4'  1NZ   1CE   1HE3
ADD  DIHE 2C4'  1NZ   1CE   1CD
ADD  IMPR 2C4   1NZ   2C4'  2H15
END


! topology of PLP in Z2 form
!---------------------------------

RESI PLP
GROUP
ATOM P    TYPE=P    CHARGE= 1.361800 END!                 H18
ATOM C5'  TYPE=CT   CHARGE= 0.299200 END!                  |
ATOM C4'  TYPE=CD   CHARGE= 0.431100 END!              H16-C2'-H17
ATOM C2'  TYPE=CT   CHARGE=-0.524300 END!          (-)O3     /
ATOM N1   TYPE=NC   CHARGE=-0.232500 END!              \    /
ATOM C2   TYPE=CA   CHARGE= 0.274000 END!    HZ1        C3--C2
ATOM C3   TYPE=CA   CHARGE= 0.443500 END!    |         //    \\
ATOM O3   TYPE=OH   CHARGE=-0.672300 END! ---NZ= C4'--C4       N1--H22
ATOM C4   TYPE=CA   CHARGE=-0.291300 END!   (+)    |     \ __ /
ATOM C5   TYPE=CA   CHARGE= 0.106000 END!       H15    C5--C6
ATOM C6   TYPE=CA   CHARGE=-0.315500 END!              /    \
ATOM OP1  TYPE=O2   CHARGE=-0.887600 END!         H13-C5'   H20
ATOM OP2  TYPE=O2   CHARGE=-0.887600 END!     (2-)    /  \
ATOM OP3  TYPE=O2   CHARGE=-0.887600 END!   OP3=P--OP4    H14
ATOM OP4  TYPE=OS   CHARGE=-0.560600 END!      /\
ATOM H13  TYPE=H1   CHARGE=-0.007600 END!    OP2 OP1
ATOM H14  TYPE=H1   CHARGE=-0.007600 END!
ATOM H15  TYPE=H4   CHARGE= 0.166400 END!
ATOM H16  TYPE=HC   CHARGE= 0.159600 END
ATOM H17  TYPE=HC   CHARGE= 0.159600 END
ATOM H18  TYPE=HC   CHARGE= 0.159600 END
ATOM H20  TYPE=HA   CHARGE= 0.196500 END
```

```
ATOM H22  TYPE=H    CHARGE= 0.368400 END
BOND P     OP1
BOND P     OP2
BOND P     OP3
BOND P     OP4
BOND C5'   C5
BOND C5'   OP4
BOND C5'   H13
BOND C5'   H14
BOND C4'   C4
BOND C4'   H15
BOND C2'   C2
BOND C2'   H16
BOND C2'   H17
BOND C2'   H18
BOND N1    C2
BOND N1    C6
BOND N1    H22
BOND C2    C3
BOND C3    O3
BOND C3    C4
BOND C4    C5
BOND C5    C6
BOND C6    H20
IMPR  C3  C5  C4  C4'
IMPR  C4  C6  C5  C5'
IMPR  C5  N1  C6  H20
IMPR  C6  C2  N1  H22
IMPR  N1  C3  C2  C2'
IMPR  C2  C4  C3  O3
DONO H22  N1
ACCE OP1  P
ACCE OP2  P
ACCE OP3  P
ACCE O3   C3
END

PRES COVA
DELE ATOM 1HZ2  END
DELE ATOM 1HZ3  END
MODI ATOM 1CD   TYPE=C8   CHARGE=-0.274600 END
MODI ATOM 1HD2  TYPE=HC   CHARGE= 0.056500 END
MODI ATOM 1HD3  TYPE=HC   CHARGE= 0.056500 END
MODI ATOM 1CE   TYPE=C8   CHARGE= 0.450800 END
MODI ATOM 1HE2  TYPE=HP   CHARGE=-0.029700 END
```

```
MODI ATOM 1HE3  TYPE=HP   CHARGE=-0.029700 END
MODI ATOM 1NZ   TYPE=NA   CHARGE=-0.536400 END
MODI ATOM 1HZ1  TYPE=H    CHARGE= 0.379000 END
ADD  BOND 1NZ   2C4'
ADD  ANGL 1CE   1NZ   2C4'
ADD  ANGL 1HZ1  1NZ   2C4'
ADD  ANGL 1NZ   2C4'  2C4
ADD  ANGL 1NZ   2C4'  2H15
ADD  DIHE 2C5   2C4   2C4'  1NZ
ADD  DIHE 2C3   2C4   2C4'  1NZ
ADD  DIHE 2C4   2C4'  1NZ   1CE  MULT 2
ADD  DIHE 2H15  2C4'  1NZ   1CE
ADD  DIHE 2C4   2C4'  1NZ   1HZ1
ADD  DIHE 2H15  2C4'  1NZ   1HZ1
ADD  DIHE 2C4'  1NZ   1CE   1HE2
ADD  DIHE 2C4'  1NZ   1CE   1HE3
ADD  DIHE 2C4'  1NZ   1CE   1CD
ADD  IMPR 2C4   1NZ   2C4'  2H15
ADD  IMPR 2C4'  1CE   1NZ   1HZ1
END


! topology of PLP in Z1 form
!--------------------------------

RESI PLP
GROUP
ATOM P    TYPE=P    CHARGE= 1.311600 END!                  H18
ATOM C5'  TYPE=CT   CHARGE= 0.311600 END!                   |
ATOM C4'  TYPE=CD   CHARGE= 0.431100 END!               H16-C2'-H17
ATOM C2'  TYPE=CT   CHARGE=-0.524300 END!          (-)O3     /
ATOM N1   TYPE=NC   CHARGE=-0.232500 END!               \   /
ATOM C2   TYPE=CA   CHARGE= 0.274000 END!   HZ1         C3--C2
ATOM C3   TYPE=CA   CHARGE= 0.443500 END!    |         //    \\
ATOM O3   TYPE=OH   CHARGE=-0.672300 END! ---NZ= C4'--C4       N1--H22
ATOM C4   TYPE=CA   CHARGE=-0.291300 END!   (+)  |      \ __  /
ATOM C5   TYPE=CA   CHARGE= 0.106000 END!        H15    C5--C6
ATOM C6   TYPE=CA   CHARGE=-0.315500 END!              /    \
ATOM OP1  TYPE=OH   CHARGE=-0.668900 END!          H13-C5'   H20
ATOM OP2  TYPE=O2   CHARGE=-0.759300 END!      (-)    /  \
ATOM OP3  TYPE=O2   CHARGE=-0.759300 END!   OP3=P--OP4    H14
ATOM OP4  TYPE=OS   CHARGE=-0.517500 END!      /\
ATOM H13  TYPE=H1   CHARGE= 0.028800 END!    OP2 OP1
ATOM H14  TYPE=H1   CHARGE= 0.028800 END!         |
ATOM H15  TYPE=H4   CHARGE= 0.166400 END!        H19
```

```
ATOM H16  TYPE=HC   CHARGE= 0.159600 END
ATOM H17  TYPE=HC   CHARGE= 0.159600 END
ATOM H18  TYPE=HC   CHARGE= 0.159600 END
ATOM H20  TYPE=HA   CHARGE= 0.196500 END
ATOM H22  TYPE=H    CHARGE= 0.368400 END
ATOM H19  TYPE=HO   CHARGE= 0.446600 END
BOND P     OP1
BOND P     OP2
BOND P     OP3
BOND P     OP4
BOND C5'   C5
BOND C5'   OP4
BOND C5'   H13
BOND C5'   H14
BOND C4'   C4
BOND C4'   H15
BOND C2'   C2
BOND C2'   H16
BOND C2'   H17
BOND C2'   H18
BOND N1    C2
BOND N1    C6
BOND N1    H22
BOND C2    C3
BOND C3    O3
BOND C3    C4
BOND C4    C5
BOND C5    C6
BOND C6    H20
BOND OP1   H19
IMPR  C3  C5  C4  C4'
IMPR  C4  C6  C5  C5'
IMPR  C5  N1  C6  H20
IMPR  C6  C2  N1  H22
IMPR  N1  C3  C2  C2'
IMPR  C2  C4  C3  O3
DONO H22  N1
DONO H19  OP1
ACCE OP2  P
ACCE OP3  P
ACCE O3   C3
END

PRES COVA
DELE ATOM 1HZ2  END
```

```
DELE ATOM 1HZ3  END
MODI ATOM 1CD   TYPE=C8   CHARGE=-0.274600 END
MODI ATOM 1HD2  TYPE=HC   CHARGE= 0.056500 END
MODI ATOM 1HD3  TYPE=HC   CHARGE= 0.056500 END
MODI ATOM 1CE   TYPE=C8   CHARGE= 0.450800 END
MODI ATOM 1HE2  TYPE=HP   CHARGE=-0.029700 END
MODI ATOM 1HE3  TYPE=HP   CHARGE=-0.029700 END
MODI ATOM 1NZ   TYPE=NA   CHARGE=-0.536400 END
MODI ATOM 1HZ1  TYPE=H    CHARGE= 0.379000 END
ADD  BOND 1NZ   2C4'
ADD  ANGL 1CE   1NZ   2C4'
ADD  ANGL 1HZ1  1NZ   2C4'
ADD  ANGL 1NZ   2C4'  2C4
ADD  ANGL 1NZ   2C4'  2H15
ADD  DIHE 2C5   2C4   2C4'  1NZ
ADD  DIHE 2C3   2C4   2C4'  1NZ
ADD  DIHE 2C4   2C4'  1NZ   1CE  MULT 2
ADD  DIHE 2H15  2C4'  1NZ   1CE
ADD  DIHE 2C4   2C4'  1NZ   1HZ1
ADD  DIHE 2H15  2C4'  1NZ   1HZ1
ADD  DIHE 2C4'  1NZ   1CE   1HE2
ADD  DIHE 2C4'  1NZ   1CE   1HE3
ADD  DIHE 2C4'  1NZ   1CE   1CD
ADD  IMPR 2C4   1NZ   2C4'  2H15
ADD  IMPR 2C4'  1CE   1NZ   1HZ1
END

}

SET ECHO=TRUE END
```

```
! force field parameters for PLP; X Chen & T Simonson, 2022
! this file: PLP.prm

BONDS
OS  P   525.0    1.61
OH  P   525.0    1.61
H   NC  434.0    1.010
CA  CD  317.0    1.510
CD  H4  350.1    1.083
CD  NA  477.0    1.343
C8  NA  337.0    1.463
CD  N2  488.0    1.335
CD  O   570.0    1.229

THETAS
O2  P   OH   140.0  108.23
HO  OH  P    140.0  108.5
CA  CT  OS   50.0   109.50
CA  CT  H1   50.0   109.50
HA  CA  NC   35.0   116.0
CA  CA  NC   70.0   124.0
CA  NC  CA   70.0   117.0
CA  NC  H    50.0   121.5
CT  CA  NC   70.0   116.0
CA  CA  CD   70.0   120.00
CA  CD  H4   50.0   120.00
CA  CD  NA   70.0   120.00
CD  NA  H    50.0   120.00
H4  CD  NA   50.0   120.00
CD  NA  C8   70.0   120.00
C8  NA  H    50.0   120.00
C8  C8  NA   80.0   111.20
HP  C8  NA   50.0   109.50
CA  CD  N2   70.0   120.00
H4  CD  N2   50.0   120.00
CD  N2  C8   70.0   117.00
HP  C8  N2   50.0   109.50
CA  CD  O    80.0   120.40
H4  CD  O    50.0   120.00

PHI
!
!Schiff base dihedral: final parameters
CA  CD  N2  C8   3.0    2   180.0   !Schiff base, N form
CA  CD  N2  C8   2.0    1   0.0     !Schiff base, N form
CA  CD  NA  C8   1.0    2   180.0   !Schiff base, Z form
CA  CD  NA  C8   2.0    1   0.0     !Schiff base, Z form
!Schiff base; initial, QM-based parameters
!CA  CD  N2  C8   10.0    2   180.0 !Schiff base, N form
!CA  CD  N2  C8   5.0    1   0.0    !Schiff base, N form
!CA  CD  NA  C8   2.0    2   180.0  !Schiff base, Z form
!CA  CD  NA  C8   4.0    1   0.0    !Schiff base, Z form
!
!Other dihedral parameters
X   CA  CD  X    1.0    2   180.0
```

H4 CD N2 C8 1.0 2 180.0
X C8 NA X 0.0 3 0.0
CA CD NA H 1.0 2 180.0
H4 CD NA C8 1.0 2 180.0
H4 CD NA H 1.0 2 180.0

IMPHI
CA CA CA CD 1.1 2 180.0
CA CA CA CT 1.1 2 180.0
CA NC CA HA 1.1 2 180.0
CA CA NC H 1.1 2 180.0
NC CA CA CT 1.1 2 180.0
CA CA CA OH 1.1 2 180.0
CA NA CD H4 1.1 2 180.0
CD C8 NA H 1.1 2 180.0
CA N2 CD H4 1.1 2 180.0
CD C8 N2 H 1.1 2 180.0
CA O CD H4 1.1 2 180.0
